# Electrochemical Immunosensor Using Electroactive Carbon Nanohorns for Signal Amplification for the Rapid Detection of Carcinoembryonic Antigen

**DOI:** 10.3390/bios13010063

**Published:** 2022-12-30

**Authors:** Angélica Domínguez-Aragón, Erasto Armando Zaragoza-Contreras, Gabriela Figueroa-Miranda, Andreas Offenhäusser, Dirk Mayer

**Affiliations:** 1 Institute of Biological Information Processing, Bioelectronics (IBI-3), Forschungszentrum Jülich GmbH, 52428 Jülich, Germany; 2 Centro de Investigación en Materiales Avanzados, S.C. Miguel de Cervantes 120, Complejo Industrial Chihuahua, Chihuahua 31136, Mexico

**Keywords:** electrochemical immunosensor, carcinoembryonic antigen, carbon nanohorns, redox-tag

## Abstract

In this work, a novel sandwich-type electrochemical immunosensor was developed for the quantitative detection of the carcinoembryonic antigen, an important tumor marker in clinical tests. The capture antibodies were immobilized on the surface of a gold disk electrode, while detection antibodies were attached to redox-tagged single-walled carbon nanohorns/thionine/AuNPs. Both types of antibody immobilization were carried out through Au-S bonds using the novel photochemical immobilization technique that ensures control over the orientation of the antibodies. The electroactive SWCNH/Thi/AuNPs nanocomposite worked as a signal tag to carry out both the detection of carcinoembryonic antigen and the amplification of the detection signal. The current response was monitored by differential pulse voltammetry. A clear dependence of the thionine redox peak was observed as a function of the carcinoembryonic antigen concentration. A linear detection range from 0.001–200 ng/mL and a low detection limit of 0.1385 pg/mL were obtained for this immunoassay. The results showed that carbon nanohorns represent a promising matrix for signal amplification in sandwich-type electrochemical immune assays working as a conductive and binding matrix with easy and versatile modification routes to antibody and redox tag immobilization, which possesses great potential for clinical diagnostics of CEA and other biomarkers.

## 1. Introduction

Cancer is a life-threatening disease with worldwide significance for the healthcare systems and a huge economic impact. Tumor biomarkers are important tools for the detection of cancer diseases, which either originate from tumor cells or emerge from the organism as a response to it. Alterations of their concentration in the body fluids may correlate qualitatively or quantitatively with the presence of cancer cells and therefore possess important clinical value for the early detection and diagnosis of the cancer diseases and thus the prognosis of the patient [1]. In fact, some biomarkers have been routinely used in clinical diagnosis including carcinoembryonic antigen (CEA), alpha-fetoprotein, prostate-specific antigen, carbohydrate antigen 125, carbohydrate antigen 153, carbohydrate antigen 199, and so on [2]. Among them, CEA, which is a set of glycoproteins of great relevance for cell adhesion during fetal development, has been considered a common cancer biomarker in clinical diagnosis since its expression declines after birth. CEA overexpression in blood serum in adult humans is usually related to the presence or progression of different types of cancer such as colorectal, liver, breast, ovarian or lung. In addition, CEA levels can also be monitored during chemotherapy to assess the progress and result of the treatment [3]. In healthy individuals, the concentration of CEA in blood serum should be less than 3 ng/mL [4]. Therefore, the development of simple and accurate methods for ultrasensitive monitoring of CEA is of great importance to help detect the presence of cancerous tumors without the need to use invasive or costly methods.

Immunoassays are important analytical techniques based on specific antigen-antibody interactions, which are widely used in clinical diagnosis. Numerous conventional immunoassays for CEA determination, including enzyme-linked immunosorbent assay (ELISA) [5], fluorescence immunoassay [1], and electrochemical immunoassay [3], have been reported. For instance, electrochemical immunosensors are considered being promising tools due to their simple instrumentation, portability, high sensitivity, low cost, and fast response. In conventional sandwich-type immunosensors, the detection antibodies are usually labeled with a peroxidase enzyme to generate the amperometric detection signal [6,7]. Although this method is sensitive and very useful, it has been shown that the biological tag can be replaced with nanomaterials for signal conversion and amplification [3]. 

Single-walled carbon nanohorns (SWCNH) are an emerging class of semiconducting nanocarbons, similar to carbon nanotubes, composed of single-layer of graphene wrapped to nanosized sheaths. SWCNH form spherical aggregates with a diameter of around 80–100 nm with different morphologies, such as dahlia, bud, and seed structures, rather than dispersing separately [8]. These unique morphologies provide special properties, such as a large surface area, small size, numerous internal nanospaces, high conductivity, and mechanical strength, making them ideal nanomaterials for application in electrochemical sensors. In addition, SWCNH can be functionalized by chemical oxidation to obtain a highly hydrophilic material and to multiply the number of binding sites on the surface for the coupling with biomolecules, metal particles, etc [9]. SWCNH can be directly used in electrochemical sensing for electrode preparation due to their superior conductivity or they can be adopted as a signal tag after their decoration with redox groups [10]. Due to the high surface area, a large number of signal molecules can be attached to the SWCNHs to facilitate strong signal amplification and consequently low detection limits.

In this work, a sandwich-type electrochemical immunosensor was developed. The capture antibodies (AntiCEA_1_) were immobilized on the surface of a gold disk electrode, while detection antibodies (AntiCEA_2_) were tethered to a nanocomposite based on SWCNHs functionalized with thionine (Thi) and gold nanoparticles (AuNPs) (SWCNH/Thi/AuNPs). Both types of antibody immobilization were carried out using the photochemical immobilization technique (PIT). In this technique, antibodies are exposed to UV irradiation, which leads to selective photoreduction of the disulfide bonds in specific cysteine-cysteine/tryptophan triads (Cys-Cys/Trp) [11]. The breaking of these Cys-Cys bonds produced free thiol groups, which can interact with the proximal gold surface, resulting in a covalent bonding of the antibody. Besides, PIT ensures control over the orientation of immobilized Abs, with their binding sites exposed to the solution phase and accessible for antigen coupling [12]. The main advantage of this immunosensing system is the reduction of the fabrication time that the PIT method provided; the immobilization of the antibodies required only 15 min. Meanwhile, the PIT technique used in the present work does not require any additional surface modification steps at the electrode, which decreased the total fabrication time to only 2.25 h, notably less than that of other reported assays. 

Thionine was used as a redox tag for the amperometric detection scheme and a multitude of these redox molecules was attached to the large surface area of the SWCNH. The detection was carried out through differential pulse voltammetry (DPV), where the change in the current intensity of the redox peak of thionine was related to the concentration of the biomarker CEA. The SWCNH were thus employed as conductive, high surface area but still small binding matrix for the attachment of AuNP-antibody entities and redox tags. 

## 2. Materials and Methods

### 2.1. Characterization

Cyclic voltammetry (CV) and differential pulse voltammetry (DPV) were measured on a multichannel potentiostat (CHI1030B, CH Instruments, Inc. Austin, USA.) with a three-electrode configuration. While electrochemical impedance spectroscopy was measured on a BioLogic potentiostat (SP-300, BioLogic Systems, Grenoble, France. The gold electrode (Au-disk, 2 mm diameter) was used as the working electrode, and a Pt wire and saturated Ag/AgCl electrode were used as the counter electrode and reference electrode, respectively. All potentials in this work are quoted with respect to the potential of the Ag/AgCl reference electrode.

The morphology and energy-dispersive X-ray spectroscopy (EDS) mapping was analyzed with a high-resolution Hitachi 7700 transmission electron microscope (TEM, Hitachi High-Technologies Corporation, Ibaraki, Jpan) and with a scanning electron microscope (SEM, Magellan 400, FEI, Hillsboro, OR, USA, and 1550VP, Carl. Zeiss SMT AG, Oberkochen, Germany).

### 2.2. Materials and Reagents

Potassium ferrocyanide K_4_[Fe(CN)_6_], potassium ferricyanide K_3_[Fe(CN)_6_], thionine acetate salt, gold nanoparticles (5 nm diameter), and oxidized carbon nanohorns were obtained from Sigma-Aldrich (Merck KGaA, Darmstadt, Germany). Phosphate buffer solution (PBS) (0.01 M) was prepared from sodium chloride (NaCl), potassium chloride (KCl), disodium phosphate Na_2_HPO_4_ and dipotassium phosphate (KH_2_PO_4_), AntiCEA_1_, AntiCEA_2_ and CEA were purchased from mybiosource.com.

### 2.3. Fabrication of SWCNH/Thionine/AuNPs Nanocomposite (SWCNH/Thi/AuNPs)

In this method, 2 mL of a SWCNH (I) dispersion (1 mg/mL) was mixed with 2 mL of thionine (4 mg/mL), stirring vigorously for 24 h at room temperature. The product was purified with Milli-Q water by centrifugation (12,000 rpm) to remove unbound thionine molecules. The SWCNH/Thi (II) was dispersed in 2 mL of Milli-Q water and then, 8 mL of AuNPs dispersion was added to the dispersion. The mixture was allowed to react for 48 h under magnetic stirring. Subsequently, the mixture was washed several times by centrifugation (12,000 rpm); the recovered solid was redispersed in 2 mL of 0.01 M PBS and stored at 4 °C.

### 2.4. Preparation of Detection Antibody Labeled SWCNH/Thi/AuNPs/AntiCEA_2_

AntiCEA_2_ was immobilized on SWCNH/Thi/AuNPs by covalent interaction between AntiCEA_2_ and AuNPs. Briefly, 350 µL of AntiCEA_2_ (121.42 µg/mL) was irradiated with a UV lamp (Trylight^®^, Promete Srl. Naples, Italy) for 30 s; afterward, it was mixed with 500 µL of SWCNH/Thi/AuNPs (III) by gently stirring for 15 min. The UV source consisted of two U-shaped low-pressure mercury lamps (6 W at 254 nm) in which a standard quartz cell could be easily housed. Considering the envelope geometry of the lamps and the cell proximity, the irradiation intensity used to produce the thiol group was approximately 0.3 W/cm^2^ [12].

The obtained SWCNH/Thi/AuNPs/AntiCEA_2_ (IV) nanocomposite was washed by centrifugation (12,000 rpm) with PBS to remove unbound material. Then, the product was redispersed in 500 µL of PBS 0.01 M. To avoid non-specific adsorption on the surface of the AuNPs, 350 µL of aqueous bovine serum albumin (BSA, Sigma Aldrich) solution (50 µg/mL) was added to the SWCNH/Thi/AuNPs/AntiCEA_2_, shaking gently for 1 h. Finally, the SWCNH/Thi/AuNPs/AntiCEA_2_/BSA (V) system was washed again, and the recovered material was redispersed in 500 µL of 0.01 M PBS and stored at 4 °C. For practical purposes, the term SWCNH/Thi/AuNPs/AntiCEA_2_/BSA will be referred to as the nanoprobe (NaPro). 

### 2.5. Assembly Process of the Immunosensor

First, the Au-disk was polished on a micro cloth using 0.3 µm and later 0.05 µm alumina. Then, it was electrochemically annealed by 100 cyclic voltammetry scans using H_2_SO_4_ 0.5 M at a potential sweep of 0.35 to 1.5 V at 1 V s^−1^ (VI). The CV with H_2_SO_4_ did not only clean the Au surface, but also worked as a pretreatment to improve the electroactive area of the Au electrode, helping with the sensitivity of the immunosensor. 

**Figure 1:**
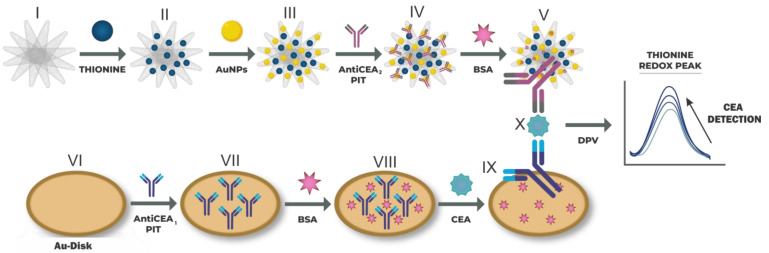
(I–V) Preparation of the nanoprobe consisting of SWCNH/Thi/AuNPs/AntiCEA_2_. (VI–X) Immobilization of the AntiCEA_2_ on the Au-disk electrode by PIT and assembly of the electrochemical immunosensor. The electrochemical detection is achieved by a dependence on the thionine redox peak as a function of the CEA concentration.

The immobilization of the AntiCEA_1_ antibody, on the surface of the gold electrode, was carried out using the photochemical immobilization technique (PIT). Briefly, 350 µL of AntiCEA_1_ (15 µg/mL) was irradiated with a UV lamp (Trylight^®^, Promete Srl) using a quartz cell for 30 s. The irradiated AntiCEA_1_ was transferred to an Eppendorf tube, and the gold electrode was immediately dipped in the solution for 15 min. Subsequently, the electrode was rinsed with PBS, obtaining Au-disk/AntiCEA_1_ (VII). Afterward, 25 μL of BSA (50 μg/mL) was deposited on the Au-disk/AntiCEA_1_ and incubated for 30 min at room temperature to avoid non-specific absorption. Subsequently, the electrode was rinsed with PBS, obtaining Au-disk/AntiCEA_1_/BSA (VIII). Then, 25 μL of CEA antigen was deposited at different concentrations and incubated for 45 min at room temperature. Afterward, the electrode was rinsed with PBS, obtaining Au-disk/AntiCEA_1_/BSA/CEA. Finally, 30 μL of NaPro was deposited and incubated for 45 min at room temperature, and the electrode was rinsed with PBS, obtaining Au-disk/AntiCEA_1_/BSA/CEA/NaPro. Figure 1 shows the assembly steps of the immunosensor.

### 2.6. CEA Biomarker Detection

The modification step-wise process of the working electrode was characterized by cyclic voltammetry (CV) and electrochemical impedance spectroscopy (EIS) in [Fe(CN)_6_]^3-/4-^ 10 mM in PBS 0.01 M. CEA detection was carried out by differential pulse voltammetry (DPV) in PBS 0.01 M at pH 7.4, monitoring the redox peak of thionine around −0.25 V.

## 3. Results

### 3.1. SWCNH/Thi/AuNPs Characterization

**Figure 2:**
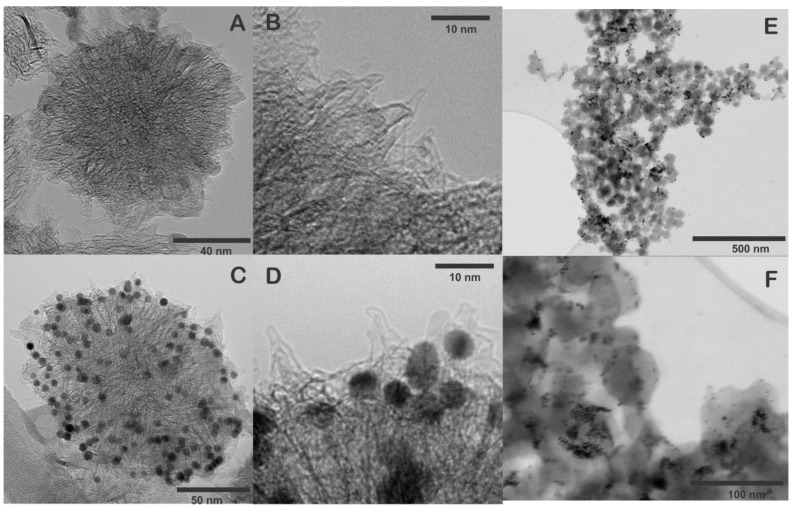
(**A**,**B**) HRTEM images of pristine SWCNH, (**C**,**D**) HRTEM images of SWCNH/Thi/AuNPs, (**E**,**F**) STEM images of SWCNH/Thi/AuNPs.

The morphology of SWCNH and SWNH/Thi/AuNPs were characterized by HRTEM and STEM. Figure 2A,B shows that a single carbon nanohorn is around 2–5 nm in diameter and 40–50 nm in length. The individual nanohorns tend to aggregate forming the typical dahlia-like nanostructure with an approximate diameter of 80–100 nm [8,13]. The STEM images of SWCNH also confirmed the dahlia-like assemblies.

Figure 2C–F show the SWCNH/Thi/AuNPs, which demonstrated the AuNPs were homogeneously distributed and anchored on the pristine SWCNH surface, providing uniform binding sites for the attachment of antibodies to the nanohorns. The average size of the AuNPs was 5–10 nm. Notably, the structure of the SWCNH was not altered during the incorporation of the AuNPs.

**Figure 3:**
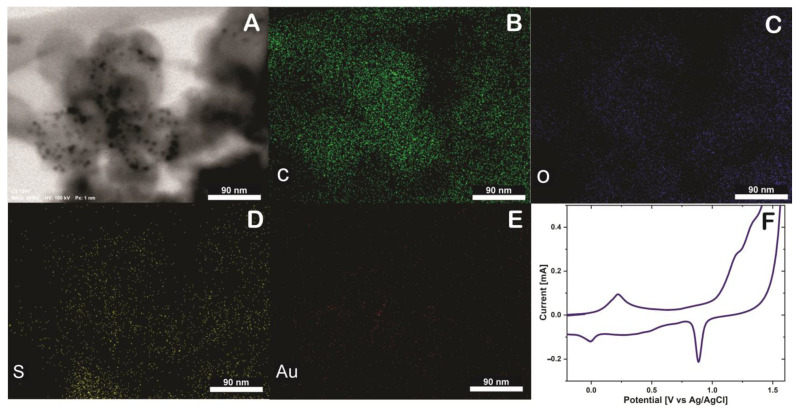
(**A**) Energy-dispersive X−ray spectroscopy (EDS) mapping of SWCNH/Thi/AuNPs, including SEM image (**B**) Carbon (C) element, (**C**) Oxigen (O) element, (**D**) Sulfur (S) element, (**E**) Gold (Au) element and (**F**) Cyclic voltammetry of glassy carbon electrode (GCE) modified SWCNH/Thi/AuNPs in H_2_SO_4_ 0.5 M at 50 mVs^−1^.

To verify the presence of thionine and AuNPs in SWCNH an EDS mapping was performed, and the elemental distribution within the SWNHs was verified using STEM-EDS (Figure 3). The SWCNH/Thi/AuNPs is mainly composed of carbon (Figure 3B), the presence of oxygen was also observed as a consequence of the oxidation treatment of SWCNH (Figure 3C), which likely contains a variety oxygen associated functional units such as hydroxyl and carboxyl groups [14]. Besides, the element sulfur was also observed (Figure 3D), which is attributed to the presence of thionine in the surface of the SWCNH since thionine contains a thiazinium group [15]. The presence of gold nanoparticles can be clearly seen on the SWCNH surface, which indicates that the nanoparticles observed by TEM and STEM are certainly gold nanoparticles, Figure 3E. These results prove that thionine and AuNPs were firmly tethered to the SWCNH with uniform distribution and cannot be washed off be rinsing.

Possible explanations for strong interaction between these components are on the one hand that thionine has a planar aromatic structure that facilitates strong π-π stacking interactions to the likewise aromatic SWCNH surface [16]. In addition to π- stacking interactions, also coupling via (electro)activated functional groups (-C=O- and COOH) of SWCNH can be involved in thionine linking via its amino-groups [17]. Additionally, the AuNPs were attached mainly to the SWCNH surface predominantly via unspecific adsorption, which could involve physisorption, π-π stacking, hydrophobic and electrostatistic interactions [18].

A glassy carbon electrode was modified with SWCNH/Thi/AuNPs and characterized by cyclic voltammetry (CV). Figure 3F shows an anodic peak around 0.21 V and a cathodic peak at 0 V, which are characteristic potentials of the reversible two-electron transfer process of thionine at acidic pH [19]. The difference between the anodic and cathodic peak is 210 mV, and the current ratio *I*_pa_/*I*_pc_ is 2.41 mA. Therefore, the thionine redox reaction can be considered as quasi-reversible process [20]. 

Noteworthy, the redox properties of thionine were not affected by the incorporation into the SWCNH. Moreover, a prominent cathodic peak was observed at approximately 0.9 V. This peak is characteristic for the reduction of gold in the reverse potential sweep after a considerable electro-oxidation in the forward sweep to potentials higher than 1.2 V, confirming the presence of the AuNPs [21]. 

The SWCNH/Thi/AuNPs nanocomposite showed distinct redox activity, supporting the incorporation of Thi on the SWCNH surface. Furthermore, it should be pointed out that the high conductivity of the SWCNH facilitated the electron transfer across this carbon material and thus the redox reaction of Thi molecules at the distal side of SWCNH, which enhanced the electrochemical current [22,23]. Likewise, the multitude of SWCNH associated AuNPs offers multiple sites for antibody immobilization, promising all in all high potential as signal tag for the fabrication of the sandwich-type immunosensor.

### 3.2. Optimization Test

**Figure 4:**
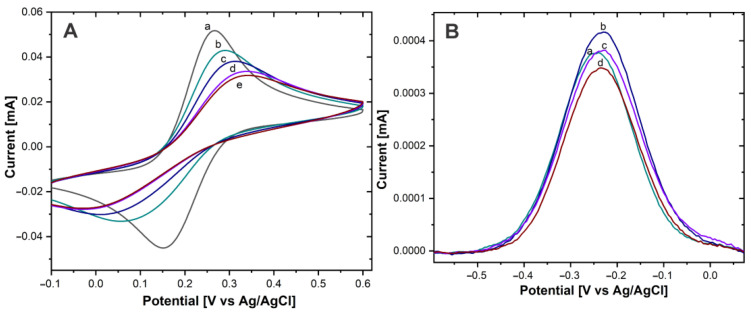
(**A**) CV of Au−disk with (a) 0, (b) 5 µg/mL, (c) 15 µg/mL, (d) 30 µg/mL, (e) 50 µg/mL of AntiCEA_1_ in Fe(CN)_6_^3-^/Fe(CN)_6_^4-^ in PBS 0.01 M at pH 7.4. (**B**) DPV of Au−disk/AntiCEA_1_/CEA/NaPro with (a) 5 µg/mL, (b) 15 µg/mL, (c) 30 µg/mL, (d) 50 µg/mL of AntiCEA_1_ in PBS 0.01 M at pH 7.4.

To verify that the immobilization of AntiCEA_1_ antibodies on the surface of the Au-disk by PIT was successful, the Au electrode was characterized by CV before and after immobilization of the antibodies, using a redox probe Fe(CN)_6_^3-^ /Fe(CN)_6_^4-^ (Figure 4).

The PIT method includes an exposure of the antibodies to UV irradiation, which leads to selective photoreduction of the typical disulfide bond of the antibodies in specific cysteine-cysteine/tryptophan triads (Cys-Cys/Trp). The breaking of these Cys-Cys bonds produces free thiol groups, which can interact with the proximal gold surface, resulting in a covalent Au-S bond between the antibody and the Au surface [12]. 

The Au-disk electrode showed well-defined anodic and cathodic peaks, due to the reversible oxidation and reduction of the solution phase Fe(CN)_6_^3-^/Fe(CN)_6_^4-^ redox molecule, with a peak-to-peak difference (Δ*E*_P_) of 112 mV (±2.16) and an anodic current intensity (*I*_P_) of 54.1 µA ± (1.87). After antibody immobilization and as the AntiCEA_1_ concentration increased, Δ*E*_P_ increased and *I*_P_ decreased, confirming that the antibodies were immobilized on the Au-disk since their covalent immobilization on the surface is acting as an insulating layer, causing slower electron transfer [24]. Besides, at the concentration of 30 µg/mL of AntiCEA_1_, the surface of the Au-disk was practically saturated, since the change of *I*_P_ from 30 to 50 µg/mL was minimal. It should be noted that the higher the concentration of immobilized capture antibodies, the higher the impact on the available electroactive surface area of the Au-disk. In other words, there could be a tradeoff between receptor density for binding the target molecule and the efficiency of the charge transfer between the electroactive surface and thionine redox probes. Hence, it is important to find an optimal immobilization concentration that leads to a receptor surface coverage at which the biosensor generates the highest analytical signal. Consequently, the electrodes were modified with different concentrations of AntiCEA_1_ and exposed to the complete detection system (CEA antigen, and the nanoprobe NaPro) at a constant concentration. 

In Figure 4B, a DPV voltammogram is shown. A redox peak around −0.22 V can be observed, which is characteristic for the Thi_ox_/Th_red_ redox couples [20]. This demonstrates that Thi was present on the NaPro and it underwent electron transfer reactions [25]. Since the amount of attached NaPro is related with the amount of CEA present in the electrode surface, Thi works as a redox tag for the electrochemical detection of CEA. The intensity of the redox peak of Thi is related to the concentration of the biomarker CEA. The highest current intensity was obtained with an AntiCEA_1_ concentration of 15 µg/mL, therefore, this concentration was chosen for the further implementation of the immunosensor.

**Figure 5:**
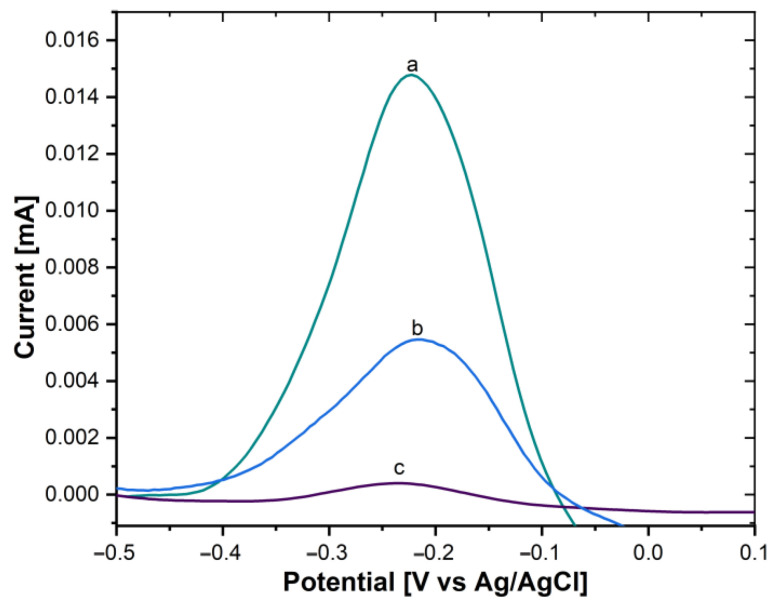
DPV of (a) GCE/SWCNH/Thi/AuNPs/0 µg/mL of AntiCEA_2_, (b) GCE/SWCNH/Thi/AuNPs/50 µg/mL of AntiCEA_2_, (c) GCE/SWCNH/Thi/AuNPs/100 µg/mL of AntiCEA_2_ in PBS 0.01 M at pH 7.4.

Moreover, the immobilization of the AntiCEA_2_ on the SWCNH/Thi/AuNPs was also carried out by the PIT. To verify the immobilization effectivity, a glassy carbon electrode (GCE) was modified with the SWCNH/Thi/AuNPs/AntiCEA_2_, testing AntiCEA_2_ concentrations of 50 and 100 µg/mL. Figure 5B shows the DPV plots of the SWCNH/Thi/AuNPs. At around −0.22 V a peak was observed that can be attributed to the redox reactions of the present thionine. The redox peak possessed a current intensity of 14.79 µA for the antibody-free SWCNH/Thi/AuNPs. This current intensity decreased after immobilization of AntiCEA_2_ to 5.44 µA (50 µg/mL) and 0.37 µA (100 µg/mL), confirming that AntiCEA_2_ was successfully immobilized. Since the concentration of 100 µg/mL significantly decreased the redox peak of thionine by blocking the charge transfer, 50 µg/mL of AntiCEA_2_ was chosen as the optimal concentration to maintain high analytical sensitivity. 

### 3.3. Electrochemical Characterization by Fabrication Steps

CV and EIS were used to corroborate the immunosensor assembly process at each modification stage and to verify the binding of the biomarker CEA and the NaPro. Both characterizations provide information on the electron transfer process and specifically, the changes in charge transfer resistance caused by anchoring the insulating biomolecules on the gold electrode.

CV tests were carried out in a Fe(CN)_6_^3-^/Fe(CN)_6_^4-^ 10 mM solution. The Δ*E*_P_ and *I*_Pa_ values were determined for Au-disk (Δ*E*_P_ = 112.4 mV ± 2.16), *I*_Pa_ = 54.11 μA ± 1.87), Au-disk/AntiCEA_1_ (Δ*E*_P_ = 335.9 mV ± 1.22, I_Pa_ = 32.7 μA ± 0.81), Au-disk/AntiCEA_1_/BSA (Δ*E*_P_ = 403 mV ± 2.04, *I*_Pa_ = 21.97 μA ± 0.49), Au-disk/AntiCEA_1_/BSA/AgCEA (Δ*E*_P_ = 498.11 mV ± 0.4, *I*_Pa_ = 16.57 μA ± 0.81), Au-disk/AntiCEA_1_/AgCEA/NaPro (Δ*E*_P_ = 376.77 mV ± 6.94, *I*_Pa_ = 29.28 μA ± 0.61).

**Figure 6:**
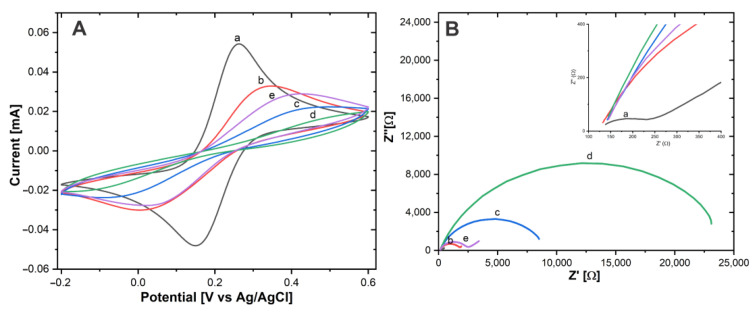
(**A**) CV and (**B**) EIS of (a) Au−disk, (b) Au−disk/AntiCEA_1_, (c) Au−disk/AntiCEA_1_/BSA, (d) Au−disk/AntiCEA_1_/BSA/CEA, (e) Au−disk/AntiCEA_1_/BSA/CEA/NaPro in 10 mM of Fe(CN)_6_^3-^/Fe(CN)_6_^4-^ in PBS 0.01 M at pH 7.4.

The height of the redox peaks consecutively decreases after the addition of AntiCEA_1_, BSA, and CEA antigen, Figure 6A. This behavior is attributed to the fact that these biomolecules do not possess conductive properties, which on the one hand do not contribute to the transport of electrons and on the other hand block the diffusion of solution-phase redox probes to the surface of the electrode [26]. In the last step, where the NaPro is incorporated, the *I*_Pa_ increased and Δ*E*_P_ decreased, indicating that the addition of the NaPro improves the electroactivity, due to the good conductive properties of the SWCNH and AuNPs, similar effect was found in previous reports [27,28]. The corresponding changes observed at each stage confirm that each component was successfully implemented in the system. 

Electrochemical impedance spectroscopy (EIS) is an effective tool to characterize the electrode-electrolyte interface properties. The charge transfer resistance (*R*_ct_) can be calculated from the semicircular section of the Nyquist plot with the axis for the real part of the impedance in EIS at low frequencies [29]. 

Fe(CN)_6_^3−^ /Fe(CN)_6_^4−^ was used as a redox couple for the EIS experiments, Figure 6B. According to the Nyquist plot, the *R*_ct_ values were Au-disk (204.65 Ω ± 5.8), Au-disk/AntiCEA_1_ (1679.19 Ω ± 15), Au-disk/AntiCEA_1_/BSA (8361.51 Ω ± 84), Au-disk/AntiCEA1/BSA/CEA (22861.61 Ω ± 116), and Au-disk/AntiCEA_1_/BSA/CEA/NaPro (2360.66 Ω ± 44). The addition of AntiCEA_1_, BSA, and CEA antigen increased the diameter of the semicircle consecutively, indicating that these biomolecules enhanced the blocking of the charge transfer at the electrode interface. Interestingly, *R*_ct_ decreased with the incorporation of the NaPro due to the highly conductive nature of the carbon nanohorns. The result of EIS coincided with the characteristics observed for CV; which demonstrates the successful implementation of a sandwich electrochemical immunosensor for the carcinoembryonic antigen detection.

### 3.4. Analytical Performance of the Immunosensor

**Figure 7:**
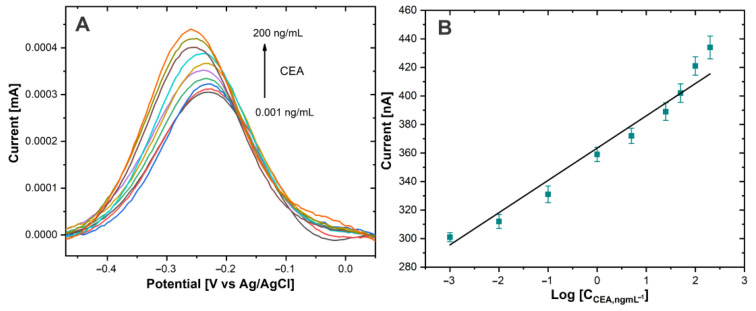
(**A**) DPV of Au−disk/AntiCEA_1_/BSA/CEA/NaPro with different concentrations of CEA in PBS 0.01 M at pH 7.4. (**B**) The linear relationship between the current peak and the log concentration of CEA.

The performance of the immunosensor for the CEA biomarker detection was investigated using DPV. The CEA antigen detection was carried out in PBS 0.01 M at pH 7.4. Figure 7A shows the immunosensor response at different concentrations of CEA. The DPV signals increased as the CEA concentration rose. 

The sensing mechanism is attributed to the Thi used as a redox tag, since a multitude of these redox molecules was attached to the large surface area of the SWCNH. The amount of attached NaPro is related with the amount of CEA present in the electrode surface due to the formation of immunocomplex between CEA and AntiCEA_2_. Therefore, the change in the current intensity of the redox peak of Thi is related to the concentration of the biomarker CEA.

The calibration curve (Figure 7B) showed a linear relationship between the current intensity of the thionine redox peak and the logarithm of the CEA concentration. The linear detection range extended from 0.001 to 200 ng/mL for CEA. The calibration curve equation was *I*_P_ (nA) = 24.726 log *C*_CEA_ (ng/mL) + 363.24 (R^2^ = 0.964) and the limit of detection was calculated to be 0.1385 pg/mL defined as the mean of the blank signal and 3 times the relative standard deviation. It should be noted that the concentration of CEA in blood serum is typically 3 ng/mL [4] in healthy individuals; therefore, the proposed immunosensor covers the medical relevant concentration range of the CEA biomarker and potentially facilitates practical application to monitor this biomarker. The promising performance of this sensor could be attributed to the high signal amplification capabilities of the highly conductive SWCNH/Thi/AuNPs and their decoration with a high number of redox active thionine. 

Compared with other previously reported methods in the literature (Table 1), our immunosensor advanced current detection technology in the combination of exhibiting a wider detection range and lower detection limits. It should also be noted that the preparation time of the previously reported systems is typically quite long because the incubation times for the immobilization of the antibodies can take several hours while here it required only 15 min thanks to the PIT activation and enhanced the immobilization via the thiol groups of the cysteines proteins. In addition, before immobilization, other methods require a modification of the electrode surface. Meanwhile, the PIT technique used in the present work does not require any additional surface modification steps, which decreases the total fabrication time to only 2.25 h, notably less than that of other reported techniques.

Besides, the immobilization of the antibodies by the PIT is very effective since it ensured control over the orientation of the immobilized Ab, with their binding sites exposed for the formation of the antigen-antibody immune complex [12,24,30]. Indeed, Funari et al. [11] investigated the immobilization and orientation of antibodies (Abs) photoactivated by PIT. In their experiments, the photoactivated antibodies were immobilized on ultrasmooth template stripped gold films and investigated by atomic force microscopy (AFM) at the level of individual molecules. They found smaller contact area and larger heights measured in the surfaces with the antibodies immobilized by PIT than the ones immobilized by physisorption. Therefore, the activated antibodies tend to be more upright compared with nonirradiated ones, thereby providing better exposure to the binding sites. The immobilization and orientation of antibodies photoactivated by PIT enhance the binding capabilities of antibody receptors, which is a critical aspect of immunosensor development because both the number and the orientation of the immobilized biomolecules are closely related to the detection efficiency of the device [31].

**Table 1:** Comparison of the proposed immunosensor with previous similar works.

Signal Tag	Fabrication Time (h)	Linear Range (ng/mL)	Detection Limit (pg/mL)	Reference
Ti_3_C_2_@CuAu-LDH	15.5	0.0001–80	0.033	[26]
PdNPs–V_2_O_5_/MWCNTs	2.5	0.0005–25	0.17	[32]
AuNP-HRP	5.32	0.01–80	2.36	[33]
NiPtAu-rGO	4	0.001–100	0.27	[34]
Au@SiO_2_/Cu_2_O	15.4	0.00001–80	0.0038	[35]
CPS@PANI@Au	8.8	0.006–12	1.56	[36]
SWCNH/Thi/AuNPs	2.25	0.001–200	0.138	This work

### 3.5. Selectivity

**Figure 8:**
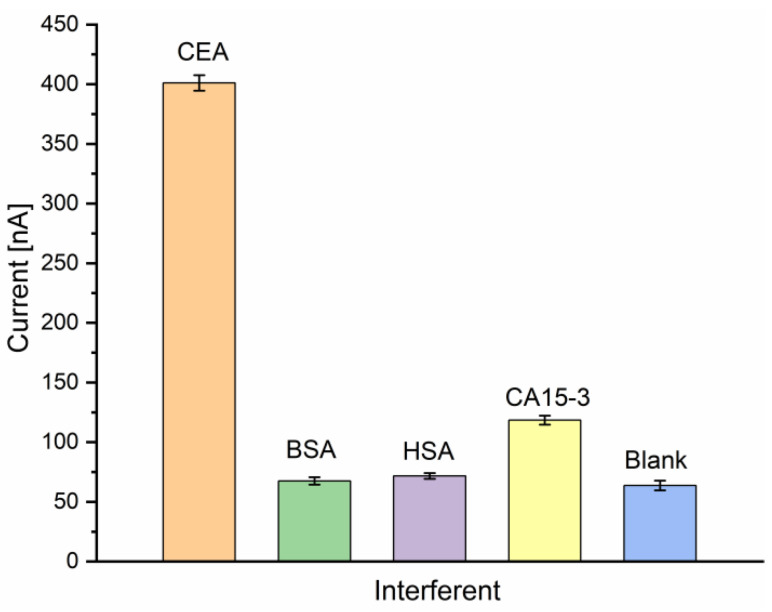
Current responses of the immunosensor to CEA (50 ng/mL) and interfering substances BSA (50 ng/mL), HSA (50 ng/mL), CA15-3 (50 U/mL) and blank in PBS 0.01 M at pH 7.4 (*n* = 3).

The high and evolutionary evolved specificity of antibodies is one advantage of immunoassays over competing biosensor concepts. To evaluate the specificity of the electrochemical immunosensor, a selectivity analysis was performed, spiking possible interfering agents such as bovine serum albumin (BSA), human serum albumin (HSA), or CA15-3 antigen to the blank sample solution (containing the nanoprobe without the presence of CEA). The tests were performed separately by incubating the Au-disk/AntiCEA_1_/BSA electrode surface in 50 ng/mL CEA, 50 ng/mL BSA, 50 ng/mL HSA, 50 U/mL CA15-3, and blank solution (0 ng/mL CEA). Although the interfering substances were applied under the same conditions as the real analyte, the response currents were much lower compared to the response toward CEA (Figure 8). This result indicates that these substances do not interfere with the target detection and the high selectivity of the antibodies was conserved during the implementation of the immunosensor, resulting in an immunosensor with excellent selectivity for CEA. 

### 3.6. Real Sample Testing

To investigate the performance of the immunosensor for detection in real clinical samples, human serum samples with known CEA concentrations were analyzed, Table 2. The standard addition method was used to corroborate electrochemical detection. CEA concentrations were calculated from the calibration curve and the tests were repeated three times for each sample. Table 2 shows the recovery (%) of the serum samples found in the range of 95 to 113%. The successful results demonstrate high accuracy and the feasibility of using the immunosensor for the electrochemical detection of CEA in real clinical samples. Therefore, the results confirm the potential of the proposed method to be implemented in the clinical field for the detection and monitoring of the carcinogenic biomarker CEA in patients. 

## 4. Conclusions

In this work, a sandwich-type electrochemical immunosensor was developed for the quantitative determination of the CEA biomarker using a signal amplification strategy based on carbon nanohorns. The fast photochemical immobilization technique (PIT) was employed for both capture and detection antibodies to tether them onto the gold electrode and the SWCNH/Thi/AuNPs, respectively, which facilitated short assay assembly times of less than three hours. The immunosensor showed a low detection limit of 0.1385 pg/mL, a linear detection range from 0.001–200 ng/mL, and high selectivity. The remarkable performance was attributed on the one hand to the antibodies being covalently bound to the gold surfaces by PIT, controlling the orientation of their active sites. On the other hand, the large surface area, high conductivity, and manifold thionine redox activity of the SWCNH/Thi/AuNP nanocomposite enhanced the amperometric sensor signal, which resulted in a high sensitivity of the device. Therefore, the proposed strategy of PIT antibody immobilization and SWCNH/Thi/AuNP nanocomposite-based signal amplification can be used as a versatile strategy for the clinical detection of the CEA biomarker and could potentially be extended for the clinical detection of other relevant biomarkers.

## Figures and Tables

**Table 2:** Results of the recovery of the immunosensor in serum samples in 0.01 M PBS.

Added CEA (ng/mL)	Found CEA (ng/mL)	Recovery (%)	% RSD (*n* = 3)
1	1.13	113.16	14.5
5	4.57	91.52	11.84
10	9.51	95.13	13.46

## Data Availability

The data presented in this study are available on request from the corresponding author.
